# Removal performance of pre- and post-dilution online hemodiafiltration using identical hemodiafilters in the same patients

**DOI:** 10.1007/s10047-022-01379-4

**Published:** 2022-12-14

**Authors:** Kazuyoshi Okada, Hiroyuki Michiwaki, Hiroaki Mori, Manabu Tashiro, Tomoko Inoue, Hisato Shima, Koji Ohshima, Jun Minakuchi, Shu Kawashima

**Affiliations:** 1 Department of Nephrology, Kawashima Hospital, 6-1 Kitasakoichibancho, Tokushima, Tokushima 770-0011 Japan; 2 Department of Clinical Engineering, Kawashima Hospital, Tokushima City, Tokushima Japan; 3 Asahi Kasei Medical Co., Ltd., 1-1-2 Yurakucho, Chiyoda-Ku, Tokyo, 100-0006 Japan

**Keywords:** Pre-dilution, Post-dilution, Online hemodiafiltration, Albumin leakage, Alpha-1-microglobulin

## Abstract

Online hemodiafiltration (OHDF) for renal replacement therapy has two modes: pre- (pre-OHDF) and post-dilution OHDF (post-OHDF). To elucidate the precise differences between the two modes, a clinical study was performed using the same polysulfone hemodiafilters in the same patients. Eight patients were treated with ABH^™^-22PA for 6 weeks: 3 weeks of pre-OHDF (with substitution volumes of 24, 36, and 48 L) and 3 weeks of post-OHDF (6, 8, and 10 L). The reduction ratios of urea, uric acid (UA), creatinine (CRE), inorganic phosphorus (iP), beta-2-microglobulin (β_2_-MG), and alpha-1-microglobulin (α_1_-MG) were evaluated. The removal amounts of β_2_-MG, α_1_-MG, and albumin were also evaluated by analyzing the spent dialysis fluids. The types and numbers of adverse events (AEs) and device malfunctions were recorded. The reduction ratios of urea, UA, CRE, iP, and β_2_-MG were comparable among all conditions, while that of α_1_-MG tended to be slightly higher in post-OHDF than in pre-OHDF. The removal amounts of α_1_-MG and albumin in pre-OHDF and post-OHDF were significantly greater with the maximum substitution volume than with the minimum volume. However, the selective removal indices, which were obtained by dividing the amount of α_1_-MG removed by the albumin level, tended to be slightly higher in pre- than in post-OHDF. No device-related AEs or device malfunctions occurred in either mode. No significant differences in inflammatory responses, evaluated by high-sensitivity C-reactive protein and interleukin-6, were observed. This study provides removal performance and safety data regarding the application of ABH-22PA for pre- and post-OHDF.

## Introduction

End-stage kidney disease (ESKD) is a major public health concern, and over 2 million people worldwide require renal replacement therapy to sustain life [1]. The three therapeutic options for renal replacement therapy are hemodialysis (HD), peritoneal dialysis (PD), and kidney transplantation. Patients with ESKD on HD or PD have a shorter life expectancy and lower quality of life (QOL) than those undergoing kidney transplantation. Over the past decades, online hemodiafiltration (OHDF) has become available and is defined as a combination of diffuse and convective solute transport using a high-flux membrane [2]. OHDF has been developed to remove middle molecules [3], which are also known as low-molecular-weight proteins (LMWPs) [4], more effectively than conventional HD. Aggressively removing LMWPs could lead to better survival rates in patients undergoing maintenance dialysis [4]. By increasing the reduction ratio of the alpha-1-microglobulin (α_1_-MG), one such middle molecule, an improvement in clinical manifestations and patients’ QOL can be expected [5]. The benefits of OHDF on all-cause mortality over conventional HD have been reported in three large randomized controlled trials [6–8]. However, a recent report showed that survival was equivalent between OHDF and super high-flux HD patients with similar albumin leakage [9].

The dilution modes for OHDF are pre-dilution (pre-OHDF) and post-dilution (post-OHDF). Post-OHDF is used in Europe and many other countries worldwide, whereas pre-OHDF is mainly used in Japan [4]. Pre-OHDF is considered advantageous because hemoconcentration is unlikely inside the filter; therefore, higher substitution volumes can be used even in patients with relatively low blood flow rates [10]. Due to hemoconcentration inside the filter, the shear stress on the blood cells activates platelets in post-OHDF [11]. Moreover, pre-OHDF may have more favorable effects on blood cells than post-OHDF because interleukin-6 (IL-6) and intercellular adhesion molecule (ICAM)-1 have been reported to be decreased only in pre-OHDF [12].

Post-OHDF has been reported to result in significantly lower all-cause mortality than conventional HD [13, 14]. Pre-OHDF was also demonstrated to have a significantly lower all-cause mortality than conventional HD in two retrospective studies [10, 14, 15]. In contrast, all-cause mortality did not significantly differ between pre- and post-OHDF [14]. Moreover, pre- and post-OHDF are known to lower the mortality in patients who receive a higher substitution volume [13, 15], while another report stated that mortality of pre-OHDF was significantly correlated with albumin leakage rather than substitution volume [9]. Therefore, differences in removal performance may affect mortality. Furthermore, if the same hemodiafilters are used in the same patients, the removal performance would still differ depending on the dilution mode [5]. Some studies focusing on evaluating the removal performances in OHDF have been reported [16–18]. However, these studies have not assessed the differences in removal performance between pre- and post-OHDF with various substitution volumes in the same hemodiafilters and patients.

In this study, the removal performance of pre- and post-OHDF under various substitution volumes was evaluated using the same hemodiafilter in the same patients. In addition, the effects of the different dilution modes on the inflammatory responses were compared.

## Materials and methods

### Ethical approval

This study was conducted in accordance with the Declaration of Helsinki of the World Medical Association. All subjects enrolled in this research provided their written informed consent, which was approved by the clinical research review board certified by the Ministry of Health, Labour and Welfare of Japan, an institutional committee on human and/or animal research that confirmed the study protocol was acceptable. This study is registered at the Japan Registry of Clinical Trials (jRCT) with the registration number jRCTs062190020.

### Patients

Eight male patients were enrolled at the start of the study. The inclusion criteria were as follows: stable maintenance OHDF therapy for at least one month; capable of obtaining QB ≥ 280 mL/min; use of hemodiafiltration with a surface area ≥ 2.2 m^2^; on OHDF for > 4 h per treatment; capable of participating in the study as an outpatient; capable of understanding the informed consent form; and age between 20 and 85 years. The exclusion criteria were as follows: patients who required blood purification therapy other than OHDF (such as PD) during the study period; those who had a medical history of anaphylaxis caused by polysulfone or polyvinylpyrrolidone (PVP); those who had participated in other clinical trials during the study period that could influence the results of this study; and disqualification from participation according to the opinion of the principal investigator.

### Study design

This was a prospective, open-label, non-randomized, single-arm study. Each patient received OHDF therapy using ABH-22PA (surface area: 2.2 m^2^ polysulfone membrane, gamma irradiation sterilization; Asahi Kasei Medical Co., Ltd. Tokyo, Japan) for 6 weeks. Each patient was administered pre-OHDF for the first 3 weeks and then post-OHDF for the next 3 weeks. The OHDF treatment condition parameters such as blood flow rate (QB; which does not include the dialysis fluid substitution flow rate (Qs)), total dialysate flow rate (total QD; which is the sum of the flow rate of the substitution fluid and dialysate flowing through the hemodiafilter), treatment time, and the number of treatments per week, were maintained during the study period for all patients at 280 mL/min, 500 mL/min, 4 h, and three times per week, respectively. The substitution volume was changed weekly in the following order: 24 L, 36 L, 48 L (pre-OHDF) and 6 L, 8 L and 10 L (post-OHDF). To assess the reduction ratios (RRs) and removal amounts, blood and dialysate drainage samples were collected during the second dialysis day of each week. In addition, during the third (pre-OHDF with 48 L substitution volume) and sixth (post-OHDF with 10 L substitution volume) weeks, high-sensitivity C-reactive protein (hs-CRP) and IL-6 levels were measured to assess inflammatory responses. The basis of the maximum substitution volumes of 48 L and 10 L for pre- and post-OHDF, respectively, is that the average substitution volumes of those in Japanese facilities are 39.9 L and 10.2 L respectively [19].

### Reduction ratio

Pre- and post-dialysis blood samples were collected within 15 min before commencing dialysis and within 15 min after dialysis completion, respectively. The blood samples were immediately centrifuged to separate the plasma, and the plasma was then used to assay the concentrations of urea, uric acid (UA), creatinine (CRE), inorganic phosphorus (iP), beta-2-microglobulin (β_2_-MG), and α_1_-MG. The RRs of urea, UA, creatinine, and iP were calculated using the following formula:

$${\text{RR }}\left( \% \right) \, = \, \left[ {\left( {{\text{Cpre }} - {\text{ Cpost}}} \right) \, / \, \left( {{\text{Cpre}}} \right)} \right] \times 100$$

where Cpre and Cpost are the concentrations of urea, UA, creatinine, or iP in peripheral blood in the pre- and post-dialysis phases, respectively.

The RRs of β_2_-MG and α_1_-MG were calculated using the following formula that includes hematocrit (HCT) correction:

$${\text{RR }}\left( \% \right) \, = \, \left\{ {1 \, - \, \left[ {{\text{HCTpre }} \times \, \left( {1 \, - {\text{ HCTpost }}/ \, 100} \right) \, \times {\text{ Cpost}}} \right]/\left[ {{\text{HCTpost }} \times \, \left( {1 \, - {\text{ HCTpre }}/ \, 100} \right) \, \times {\text{ Cpre}}} \right]} \right\} \, \times \, 100.$$

where Cpre and Cpost are the concentrations of β_2_-MG and α_1_-MG in the peripheral plasma, and HCTpre and HCTpost are the HCT values (in %) measured at the pre- and post-dialysis sessions, respectively.

### Removal amount and albumin leakage

The removal amount of β_2_-MG, α_1_-MG, and albumin leakage was calculated using the following formula:

$$\left[ {\text{Total amount of spent dialysis fluids}} \right] \, \times \, \left[ {\text{concentration of substance in dialysate drainage}} \right]$$

The partial storage method was used to store the dialysate drainage [20].

To assess the selectivity of the α_1_-MG removal for albumin leakage, the selective removal index of α_1_-MG (SRIA) was calculated [20]. The SRIA was calculated as the amount of α_1_-MG removed divided by the amount of albumin leakage in a single session using the following formula:

$$\left[ {{\text{Removal amount of }}\alpha_{1} - {\text{MG }}\left( {{\text{mg}}} \right)} \right] \, / \, \left[ {{\text{albumin leakage }}\left( {\text{g}} \right)} \right]$$

### Inflammatory responses

Blood samples were collected; they underwent the same treatment as for RR evaluation and were used to assay the concentrations of hs-CRP and IL-6.

### Adverse events and device malfunction

All adverse events (AEs) were recorded for all treatments and interdialytic days. For example, even minor events, such as a patient’s feeling of discomfort, muscle cramps requiring minimal intervention, or changing the ultrafiltration flow rate, were recorded as AEs in the case report forms. The dialysis treatment condition parameters such as flow rates of blood and dialysate, vital signs at pre- and post-dialysis, dry weights, volumes of water removed, and transmembrane pressures (TMPs) were also recorded. All device malfunctions that occurred during any of the treatment sessions were recorded as well.

### Statistical analysis

Statistical analyses for RRs, removal amounts, and albumin leakage were compared among the same dilution modes. The values for 48 L in pre-OHDF were compared in detail to those for 24 L and 36 L; similarly, the values for 10 L in post-OHDF were compared to those for 6 L and 8 L. All parameters were compared using paired *t* tests. Statistical significance was set at *P* < 0.05 (two-sided). No adjustments for multiple comparisons were performed. Statistical analyses were performed using SAS version 9.4 (SAS Institute Inc., USA).

## Results

### Patient enrollment and population analysis

Eight patients were screened for enrollment in the study, with none deemed ineligible. The patient baseline characteristics are shown in Table 1. All patients completed the OHDF therapy using ABH-22PA for 6 weeks. None were lost to follow-up, and no deaths occurred during the study. In the following evaluations, the data for all patients were defined as the safety analysis (SAA) and full analysis set (FAS) populations. However, in the sixth week only, the removal amounts of β_2_-MG, α_1_-MG, and albumin leakage were calculated using data from seven patients because the dialysate drainage samples could not be measured in one patient. The first treatment of the study began on January 13, 2020, and the last treatment was completed on February 22, 2020.

**Table 1:** Baseline characteristics

Parameters	Values
Age (mean ± SD) (years)	62.5 ± 10.7
Sex, *n* (%)	
Male	8 (100)
Female	0 (0)
Dry weight (mean ± SD) (kg)	61.43 ± 6.59
Primary kidney disease, *n* (%)	
Diabetic nephropathy	0 (0)
Chronic glomerulonephritis	5 (62.5)
Nephrosclerosis	1 (12.5)
Others	2 (25.0)

*SD* standard deviation

### Performance evaluation

The RRs of urea, UA, creatinine, iP, β_2_-MG, and α_1_-MG in the FAS population are summarized in Table 2. The RRs of urea, UA, creatinine, iP, and β_2_-MG were almost the same in pre- and post-OHDF, while the RRs of α_1_-MG tended to be slightly higher in post-OHDF. Using the same dilution mode, the larger the substitution volume, the larger the RR of α_1_-MG. The removal amounts of β_2_-MG, α_1_-MG, albumin leakage, and SRIA are summarized in Table 3. The removal amount of α_1_-MG showed a similar trend as its RR: it was slightly higher in post-OHDF, and with the same dilution treatment, the larger the substitution volume, the larger the removal amount. The removal amounts of α_1_-MG and albumin leakage of pre-OHDF at 48 L were significantly higher than those of pre-OHDF at 24 L (*P*-values were 0.017 and 0.001, respectively), and those of post-OHDF at 10 L were also significantly higher than those of post-OHDF at 6 L (*P*-values of 0.034 and 0.047, respectively). However, no significant difference was observed between pre-OHDF at 48 L and 36 L or post-OHDF at 10 L and 8 L. Moreover, no significant difference was observed in the removal amounts of β_2_-MG between the two conditions. The SRIA was almost the same when the same dilution mode was used; however, at different dilution modes, SRIA tended to be slightly higher in pre- than in post-OHDF.

**Table 2:** Reduction ratio values

	Pre-OHDF						Post-OHDF					
Substitution volume	24 L (1st week)		36 L (2nd week)		48 L (3rd week)		6 L (4th week)		8 L (5th week)		10 L (6th week)	
	Mean	± SD	Mean	± SD	Mean	± SD	Mean	± SD	Mean	± SD	Mean	± SD
Reduction ratio (%)												
Urea^1^	71.1	4.5	72.3	2.6	71.7	1.6	73.4	1.8	73.9	1.9	74.5	2.1
Uric acid^1^	77.1	4.5	78.4	3.2	78.6	3.6	79.9	3.2	79.7	3.2	79.8	3.4
Creatinine ^1^	66.4	4.2	67.0	2.0	66.9	2.2	69.0	2.3	68.8	2.2	69.3	3.5
Inorganic phosphorus^1^	54.7	6.1	57.4	4.1	55.5	5.8	55.9	6.2	55.6	5.6	58.8	6.8
Beta-2-microglobulin^2^	77.0	5.1	77.9	4.9	78.8	3.2	76.2	4.3	77.5	3.3	77.9	4.4
Alpha-1-microglobulin^2^	18.5	5.0	18.4	5.8	19.4	3.3	20.6	6.3	24.2	4.8	23.7	5.6

All values were calculated using the full analysis set (FAS) (*n* = 8)

*OHDF* online hemodiafiltration, *SD* standard deviation

^1^Reduction ratios of urea and creatinine are indicated without hematocrit correction

^2^Reduction ratios of beta-2-microglobulin and alpha-1-microglobulin are indicated with hematocrit correction

**Table 3:** Removal amount and albumin leakage

	Pre-OHDF						Post-OHDF					
Substitution volume	24 L (1st week)		36 L (2nd week)		48 L (3rd week)		6 L (4th week)		8 L (5th week)		10 L^1^ (6th week)	
	Mean	± SD	Mean	± SD	Mean	± SD	Mean	± SD	Mean	± SD	Mean	± SD
Removal amount												
Beta-2-microglobulin (mg)	216.2	63.2	219.0	37.1	231.8	56.0	240.0	63.2	236.6	54.7	256.7	60.5
Alpha-1-microglobulin (mg)	79.6	15.2	89.8	22.0	96.0^*^	15.2	103.6	25.8	109.7	19.6	120.1^*^	20.1
Albumin leakage (g)	1.94	0.40	2.27	0.44	2.46^**^	0.42	2.74	0.77	3.05	0.84	3.51^*^	0.54
SRIA^2^	41.3	4.49	39.6	6.85	39.8	8.91	38.6	5.70	37.7	9.30	34.6	6.07

All values were calculated using the full analysis set (FAS) (*n* = 8)

*OHDF* online hemodiafiltration, *SD* standard deviation, *SRIA* selective removal index of α_1_-MG

*p < 0.05 compared to the minimum substitution volume of the same dilution method

**p < 0.01 compared to the minimum substitution volume of the same dilution method

^1^All values are indicated, excluding that of one patient whose sample could not be measured (*n* = 7)

^2^Selective removal index of alpha-1-microglobulin [20]

### Inflammatory responses

The inflammatory responses in the FAS population are summarized in Table 4. In pre-OHDF, the values of hs-CRP and IL-6 after the treatment decreased compared with those before, whereas they increased in post-OHDF. However, no significant differences were observed between pre- and post-OHDF. The elevation in the mean values of hs-CRP in post-OHDF was observed in one patient who developed fever, as described in the AE section. The values were 1.71 mg/dL and 2.10 mg/dL before and after treatment, respectively. Moreover, the patient’s blood sampling date was the day after the onset of the fever. The median values of hs-CRP in pre- and post-OHDF were almost the same. Therefore, it can be concluded that the increase in the mean hs-CRP levels was due to the fever in this patient.

**Table 4:** Inflammatory responses

	Pre-OHDF		Post-OHDF	
	Before	After	Before	After
	Mean ± SD [median]	Mean ± SD [median]	Mean ± SD [median]	Mean ± SD [median]
The values before and after the treatments				
hs-CRP (mg/dL)	0.1540 ± 0.1811 [0.1155]	0.1540 ± 0.1881 [0.1065]	0.3069 ± 0.5710 [0.1155]	0.3594 ± 0.7071 [0.1185]
IL-6 (pg/mL)	9.5 ± 4.2 [8.0]	9.4 ± 3.9 [8.0]	10.3 ± 4.0 [8.0]	10.6 ± 3.9 [8.5]
The values after the treatments when the values before were defined as 100%				
	Mean ± SD [median]		Mean ± SD [median]	
hs-CRP (%)	96.53 ± 8.40 [97.28]		104.69 ± 23.99 [96.42]	
IL-6 (%)	99.38 ± 1.77 [100]		105.31 ± 18.27 [100]	

All values were calculated using the full analysis set (FAS) (*n* = 8)

*hs-CRP* high-sensitivity C-reactive protein, *IL-6* interleukin-6, *OHDF* online hemodiafiltration, *SD* standard deviation

### AE and device malfunction

Two AEs, suspected infectious gastroenteritis and fever, were recorded in two patients. These AEs improved within 1 day and were considered unrelated to ABH-22PA. No serious AEs or device malfunctions were recorded. Furthermore, complications due to elevated TMP, such as interruption of treatment, did not occur in either dilution mode.

## Discussion

The results indicated that the removal performance varied depending on the substitution volume in pre- and post-OHDF, as in previous reports [6–8, 15]. In particular, the tendency was remarkable in middle molecules, which are predominantly removed by filtration rather than diffusion.

An interesting finding of this study was that the removal performance of small-molecular-size substances, such as urea, UA, CRE, and iP, was not inferior in pre-OHDF to that in post-OHDF. Theoretically, small-molecular-size substances are predominantly removed by diffusion; therefore, their removal performance should be superior in post-OHDF. The concentration of such substances is diluted in pre-OHDF, and the concentration difference between the blood side and dialysate is less in pre- than in post-OHDF. However, in this study, the reduction ratios of small-molecular-size substances were almost the same regardless of the dilution mode. This might reflect the excellent removal performance of ABH-PA through the diffusion of small-molecular-size substances using an improved three-dimensional hollow fiber structure.

Meanwhile, post-OHDF was superior to pre-OHDF in terms of removal performance of α_1_-MG, which is predominantly removed by filtration. The removal performance of the middle molecules increases as the substitution volume increases in the same dilution mode, and comparing the dilution modes, post-OHDF is superior to pre-OHDF. This result resonates with that of previous studies [20].

For β_2_-MG, unlike α_1_-MG, no significant difference was observed in the removal performance regardless of the dilution mode or substitution volume, as with small-molecular-size substances. Therefore, β_2_-MG, with a molecular weight of 11,800, was thought to be removed mainly by diffusion rather than by filtration. This is consistent with the findings of previous reports that compared the removal performance of pre- and post-OHDF [5, 20].

As β_2_-MG is removed mainly by diffusion, it would be insufficient to attribute the improvement in mortality resulting from OHDF to β_2_-MG removal only. Instead, it is necessary to also focus on the removal of substances that have a higher molecular weight than β_2_-MG and are mainly removed by filtration. Among these substances, clinical manifestations related to dialysis are alleviated when the reduction ratio of α_1_-MG exceeds 35% [5]. When attempting to increase the reduction ratio of α_1_-MG, albumin will inevitably leak out simultaneously. This is because the Stokes radii of free α_1_-MG and albumin are similar, at 28.6 Å [21] and 35.5 Å, respectively, even though their molecular weights differ, at approximately 33,000 and 66,000, respectively. α_1_-MG has been reported to form a complex with IgA, prothrombin, and albumin within the body [22]. However, the Stokes radii of α_1_-MG combined with IgA, prothrombin, and albumin remain unknown. New types of hemodiafilters that can selectively remove α_1_-MG with low albumin leakage are under development.

In the removal performance of the selectivity of the α_1_-MG removal for albumin leakage, some differences were observed between pre- and post-OHDF. SRIA, an index of the selective removal capacity of α_1_-MG, was slightly better in pre- than in post-OHDF. However, SRIA tends to be high when the amount of albumin leakage is low, even if the removal amount of α_1_-MG is minimal. In this study, the maximum average removal amount of albumin for each dilution mode was 2.46 g in pre-OHDF with 48 L substitution volume and 3.51 g in post-OHDF with 10 L substitution volume, which were too low to assess SRIA. Therefore, the variation in substitution volume in each group was not sufficient for detailed discussion, and further studies are required to elucidate the selective removal capacity of α_1_-MG with different dilution modes.

In this study, the levels of the inflammatory markers such as hs-CRP and IL-6, both before and after treatment, demonstrated no significant difference between pre- and post-OHDF. The inflammatory response was measured during post-OHDF at a substitution volume of 10 L, and QB was 280 mL/min; thus, the ratio of filtration flow rate (Qf) to QB was approximately 15%. This value is lower than the standard ratio in EU countries. In a study regarding EU countries, more than half of post-OHDF patients were treated with a substitution volume of ≥ 20 L, and the average QB of the patients was 342 mL/min, in which case the ratio of Qf to QB was ≥ 24% [23]. Some differences in inflammatory response may be observed under conditions of higher blood concentrations. Further studies with a high Qf to QB ratio are required to confirm this assumption.

There were no device-related AEs, device malfunctions, or elevations in TMP in either the pre- or post-OHDF modes. Therefore, both pre- and post-OHDF can be regarded as sufficiently safe for treatment under the conditions evaluated in this study.

This study has some limitations. First, no female patients were involved in the study, which means that the current findings are not representative of what occurs in both males and females. Second, the findings were associated with OHDF employing only ABH-22PA. Therefore, if other instruments are employed, the results may vary. Third, there were no data on high volumes of substitution fluid as the maximum amount of substitution fluid was based on the average in Japan. Further studies employing a larger population, the use of other instruments, and higher volumes of substitution fluid are required.

This study aimed to confirm removal performance when the same hemodiafilters were used in the same patients; thus, mortality and other clinical benefits were not evaluated. Further studies are required to evaluate the relationship between substance removal, especially of middle molecules, and mortality.

## Conclusion

By using the same hemodiafilter in the same patients, the differences in removal performance between pre- and post-OHDF have become clear. Post-OHDF is superior to pre-OHDF in terms of removal performance by filtration. Meanwhile, removal performance by diffusion is almost the same, regardless of the dilution mode. Moreover, no significant difference is noted in inflammatory responses between pre- and post-OHDF.

## Data Availability

The datasets generated during and/or analysed during the current study are available from the corresponding author on reasonable request.
